# Mandibular sawing in a snail-eating snake

**DOI:** 10.1038/s41598-020-69436-7

**Published:** 2020-07-29

**Authors:** Yosuke Kojima, Ibuki Fukuyama, Takaki Kurita, Mohamad Yazid Bin Hossman, Kanto Nishikawa

**Affiliations:** 1 Department of Biology, Toho University, Funabashi, Chiba 274-8510 Japan; 2 Graduate School of Human and Environmental Studies, Kyoto University, Sakyo-ku, Kyoto 606-8501 Japan; 3 Chiba Biodiversity Center, Aoba-cho 955-2, Chuo-ku, Chiba 260-8682 Japan; 4 Research, Development and Innovation Division, Sarawak Forest Department, 93250 Sarawak, Malaysia; 5 Graduate School of Global Environmental Studies, Kyoto University, Sakyo-ku, Kyoto 606-8501 Japan

**Keywords:** Ecology, Evolution, Zoology

## Abstract

The jaws of vertebrates display a striking diversity in form and function, but they typically open and close like a trapdoor rather than sliding like a saw. Here, we report unique feeding behaviour in the blunt-headed snail-eating snake, *Aplopeltura boa* (family Pareidae), where the snake cuts off and circumvents the indigestible part (the operculum) of its prey in the mouth using long sliding excursions of one side of the mandible, while the upper jaws and the mandible on the other side maintain a stable grasp on the prey. This behaviour, which we call ‘mandibular sawing’, is made possible by extraordinarily independent movements of the jaw elements and is a surprising departure from usual feeding behaviour in vertebrates.

## Introduction

Talos, the Greek mythological inventor, invented a saw inspired by a serpent jawbone or a fish skeleton^1^, but nonetheless, the jaws of snakes or other vertebrates usually do not act like a saw due to anatomical constraint on the jaw movements (but see^2^). Because the diversity in vertebrate jaws represents modifications of the homologous apparatus, their form and function are strongly constrained by the phylogeny^3^. Advanced snakes differ from most other vertebrates in their ability to move the left and right jaws virtually independently and swallow their prey using alternate movements of the two sides of the jaws^4^. Snail-eating snakes of Southeast Asia (Pareidae) or the Neotropics (Dipsadinae) retain the unilateral mobility of the jaws, but their feeding apparatus is further modified; these snakes have lost the articulation between the upper and lower jaws in contrast to most other snakes, and this makes the lower jaws of the snail-eating snakes extensively mobile and allows the mandibles to perform independent sliding excursions^4–9^. Several species use asynchronous retractions of the mandibles to extract snails from their shells or ingest slugs^10–16^. Feeding behaviors of most species of those peculiar snakes, however, have never been described perhaps due to limited access to these tropical, nocturnal, and secretive animals.

The blunt-headed snail-eating snake, *Aplopeltura boa*, is a pareid species that feeds on snails, including operculate species^17^. Gastropodan opercula are indigestible for snakes^18^, like their shells, and therefore are potentially harmful when consumed (e.g., they may cause intestinal obstruction). Indeed, feeding experiments showed that a snail-specialist pareid did avoid eating all of three species of sympatric operculate snails, whereas it consumed most of sympatric non-operculate snail species^19^. We collected *A. boa* and a syntopic, abundant operculate snail, *Leptopoma* sp. (Cyclophoridae) in a rainforest in Borneo and observed the snake feeds on the tough prey. Also, we investigated the relative abundance of operculate and non-operculate snails in the habitat of *A. boa*.

## Results and discussion

We found that operculate snails were more abundant than were non-operculate snails: 34 and 22 individuals were encountered, respectively. Among snails of moderate size (shell width, 10–20 mm), which were often consumed by *A. boa*, operculate snails were more than 10 times as abundant as were non-operculate snails (25 and 2 individuals were encountered, respectively).

**Figure 1:**
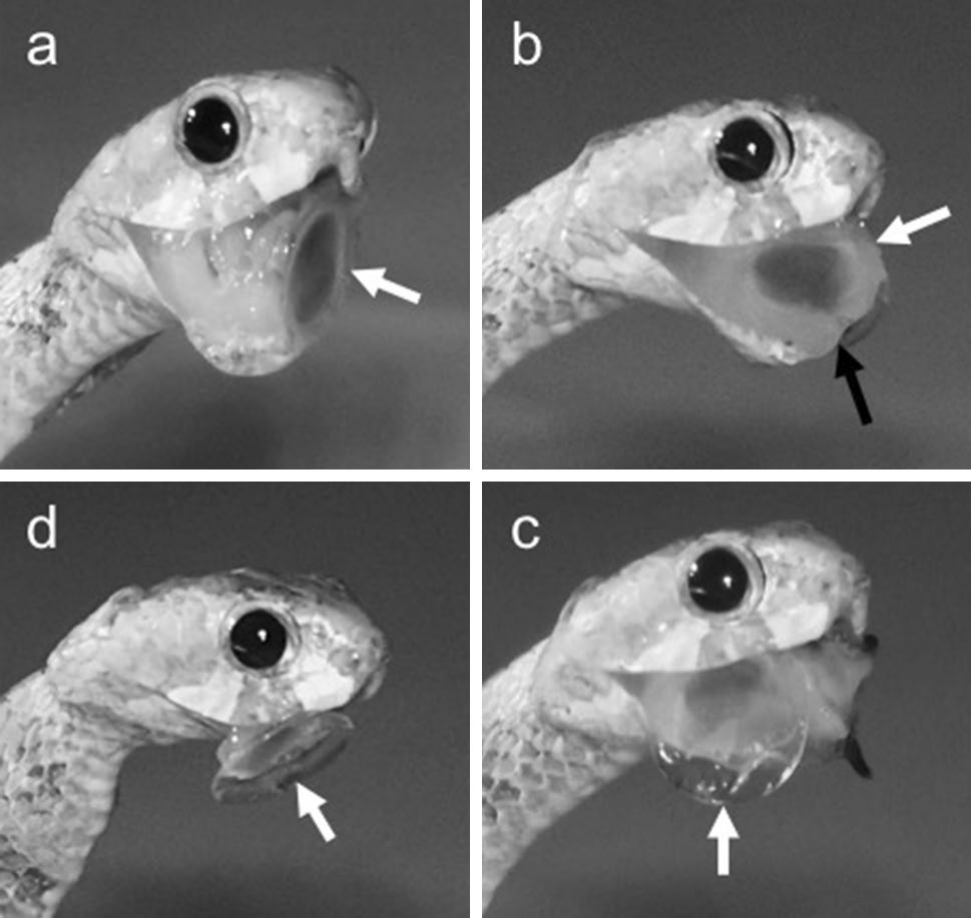
Infrared images illustrating mandibular sawing by the blunt-headed snail-eating snake, *Aplopeltura boa*. (**a)** The extracted operculate snail in the snake’s mouth. (**b**) The snail has been accurately repositioned. (**c**) The operculum being sliced off. (**d**) The soft parts of the snail being ingested while the operculum is discarded. A white and black arrow indicates the position of the operculum and the tip of the mandible, respectively. Videos showing this behavior are available with supplementary information of this article (Videos S1, S2).

Individuals of *A. boa* (n = 8) readily preyed upon *Leptopoma* sp. (n = 30) in our feeding trials. Upon capture, the snakes immediately inserted their mandibles into the aperture of the shell and then extracted the operculate soft body using the mandibles. After extraction, the snakes regurgitated the extracted snail and precisely repositioned it so that the operculum protruded out of the mouth and the junction of the operculum and the soft body came to lie along the mandible on the right (n = 24, 80%) or the left (n = 6, 20%) side. From this position, the snakes moved the side of the relevant mandible backward and forward, while the snail was held in the stable position by the upper jaws and the mandible on the other side. These mandibular movements were especially vigorous when the mandible was being retracted. Six to 51 (median, 14) strokes of these sliding movements resulted in the removal of the operculum (Fig. 1, Supplementary Information, Videos S1, S2). Snakes consumed solely the soft part of the snails, while discarding the shell and the operculum (n = 30, 100%). Discarded opercula retained little soft tissue. The duration of extraction, repositioning, and sawing processes ranged 36–274 (median, 96), 9–459 (median, 66), and 15–428 (median, 30) seconds, respectively (Supplementary Information, Table S1). Thus, the snakes spent considerable time for handling of the extracted snail (reposition and sawing). The sequence of feeding was highly consistent among all cases, and the routine operculum-removing behaviour presumably allows *A. boa* to regularly consume the otherwise hazardous prey, which is abundant in its habitat.

**Figure 2:**
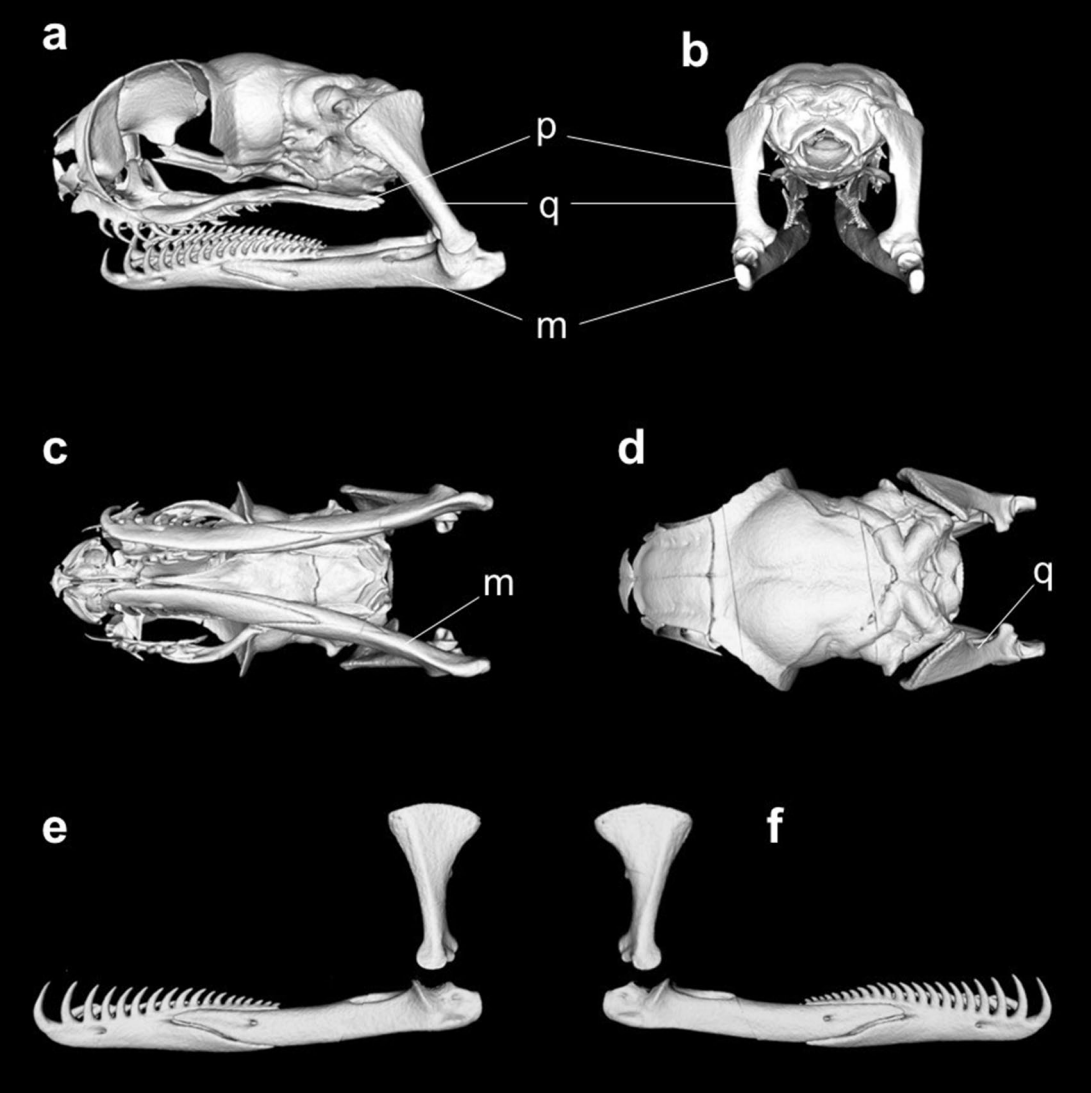
Feeding apparatus of the blunt-headed snail-eating snake, *Aplopeltura boa*. CT images of the skull and the jaws from left lateral (**a**), posterior (**b**), ventral (**c**), and dorsal (**d**) views and the quadrates and the mandibles on the left (**e**) and right (**f**) sides. m, mandible; p, pterygoid; q, quadrate. These images are from the specimen KUHE59285.

**Figure 3:**
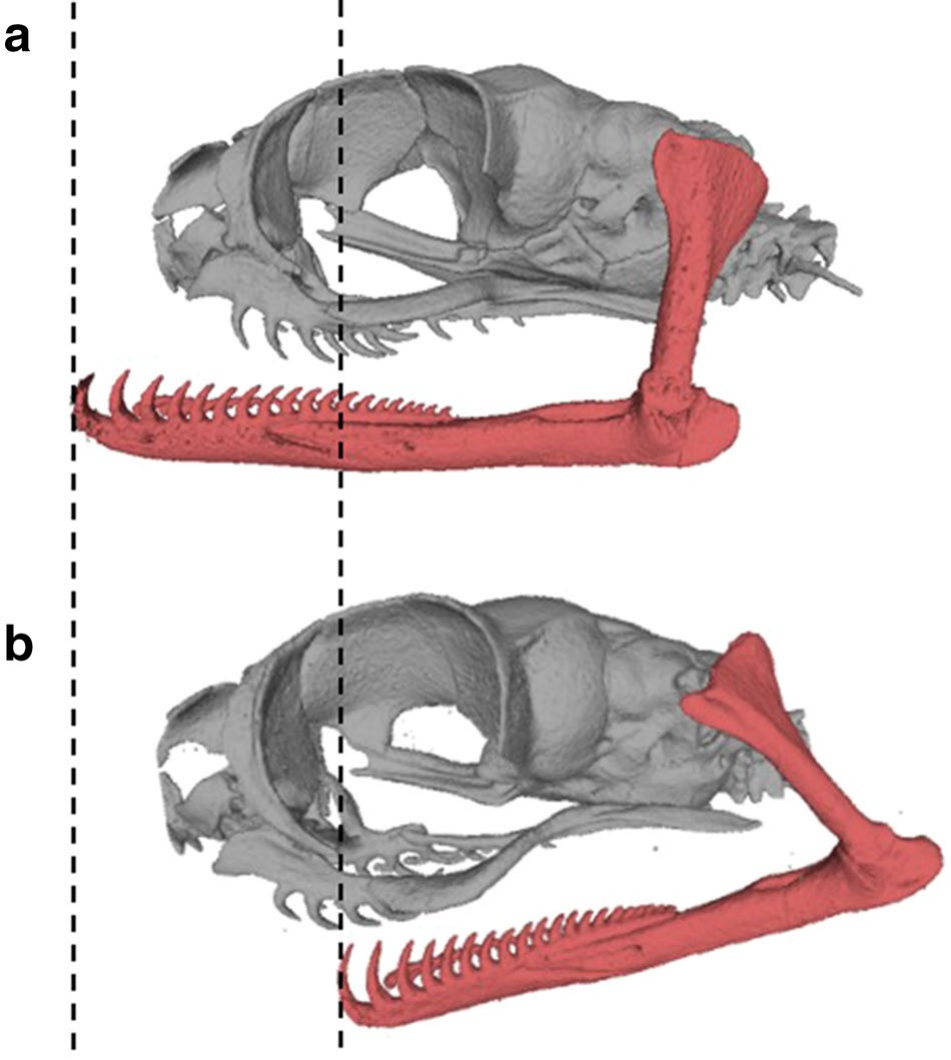
Movements of the lower jaw in the blunt-headed snail-eating snake, *Aplopeltura boa*. CT images of the skull and the jaws from left lateral view with the mandible protracted (**a**) and slightly retracted (**b**). These images and the videos on feeding behaviour demonstrate that the mandibles slide about half the length of the skull. These mandibular movements can be performed unilaterally. Images (**a**) and (**b**) represent the specimen SRC01008 and SRC01009, respectively.

The feeding apparatus of *A. boa* is illustrated in Fig. 2. *Aplopeltura boa* exhibits a set of derived morphological features known in other pareids and dipsadines, including short snout, short pterygoids, reduced supratemporals, long mandibles, and comb-like mandibular teeth. The skull of *A. boa* is short and tall, in which the snout is very short, and the orbits are exceptionally large. The pterygoids are greatly shortened, and their posterior ends are completely detached from the quadrato-mandibular joint. The quadrates are remarkably long and stout, extending ventrally rather than ventrolaterally. The mandibles are long and carry dense teeth, which are more robust than the maxillary teeth. The size and interval of mandibular teeth gradually change along the mandible; the anterior teeth are larger and sparser. The lower jaw unit (the mandible and the quadrate) is L-shaped, and the mandible travels anteroposteriorly when the quadrate swings backward and forward, as anticipated or observed in other pareids or dipsadines. This mechanism enables independent, substantial anteroposterior excursions of the mandible (Fig. 3), which is used for the extraction and the sawing processes during feeding on the operculate snails. The elongated quadrates are likely to contribute to long mandibular excursion^18^.

Most of > 3,700 species of snakes swallow their prey whole, and prey-breaking behaviours are known only from a few species that feed either on crabs^20–23^ or termites^24–26^, which may be relatively easily broken into segments. These snakes grasp their arthropod prey with their jaws and break it apart usually using the movements of the head or the trunk. In contrast, *A. boa* cuts its mollusk prey using independent movements of the lower jaw. The mandibular sawing is, therefore, a surprising evolutionary solution for the limbless animals to utilize new food. This dexterous behaviour is especially surprising given that few other vertebrates, if any, are able to sever food in the mouth using unilateral sliding movements of a jaw element like *A. boa*. The evolutionary invention of sawing was evidently made possible by the unique feeding mechanism in the snail-eating snakes. Extensive mobility of the mandibles is a convergent trait in the two distinct lineages of snakes (pareids and dipsadines) that feed on slugs or snails, suggesting it is an adaptation to feeding on their slippery prey^4–9^. It is likely that acquisition of the free mandibular apparatus promoted the subsequent evolution of the novel behaviour and has resulted in functional versatility of the free-moving jaw elements.

Most pareids and some dipsadines have a larger number of teeth on the right mandible than on the left as feeding specialization to extract dextral (clockwise-coiled) snails^13,15^. There is a cline in the degree of the dentitional asymmetry in correlation with diets, where snail-specialist species have highly asymmetrical mandibles, whereas slug-specialist species have symmetrical mandibles^13,15^. However, *A. boa* is an exception of this pattern because individuals exhibit only weak mandibular asymmetry despite its snail diet^13^. In the phylogeny, *A. boa* is nested within the derived clade with mandibular asymmetry^27^, suggesting the presence of additional selective forces toward the mandibular symmetry in this species. By showing the additional role of its mandibles (cutting the prey), our results suggest functional trade-offs in *A. boa* (typical comb-like teeth in snail-eating snakes are expected to facilitate extraction by providing a firm grip on the prey but probably are not optimal to cut the prey tissue), highlighting the importance of behavioural studies to understand selective forces on functional units.

## Methods

This study was conducted in the Gunung Mulu National Park, Sarawak, Malaysia (4.0440°N, 114.8144°E). We collected *A. boa* and *Leptopoma* sp. by visual search and brought them to the Research Centre in the national park for the feeding trials. All but three snakes that were used for other studies were released at the site of capture after trials.

Feeding trials were conducted between 2100 and 0400 h. We fed *A. boa* with *Leptopoma* sp. and video-recorded feeding events with infrared video cameras (HC-VX985M, Panasonic; FDR-AX30, Sony).

We conducted census survey on snails by walking the habitats of *A. boa* carefully looking for snails and counting all snails larger than 5 mm in shell width. This survey was conducted on two nights in August and December, respectively.

We conducted micro CT-scanning on six specimens of *A. boa* held at Kyoto University or Sarawak Forest Department (specimen nos: KUHE27025, KUHE56016, KUHE57425, KUHE59285, SRC01008, and SRC01009).

All procedures followed the Animal Experiment Guideline of Kyoto University and were approved by the ethical review committee of the Graduate School of Human and Environmental Studies of Kyoto University and the Research, Development and Innovation Division of the Sarawak Forest Department (approval no. 30-A-7). All fieldwork was permitted by the State Government of Sarawak, the Sarawak Forest Department, and the Gunung Mulu National Park (permission nos. (133)JHS/NCCD/600–7/2/107, WL72/2018, WL103/2018, and WL68/2019).

## Supplementary information

Supplementary Information.

Supplementary Video S1.

Supplementary Video S2.
