# Supplementary material for: Mandibular sawing in a snail-eating snake
Source: Sci Rep. 2020 Jul 29;10:12670. doi: 10.1038/s41598-020-69436-7 (PMC7391773; doi:10.1038/s41598-020-69436-7)
Supplement: Supplementary file 1 — Supplementary Information. [file 41598_2020_69436_MOESM1_ESM.pdf]

Supplementary information to

Mandibular sawing in a snail-eating snake

Yosuke Kojima[1*], Ibuki Fukuyama[2], Takaki Kurita[3], Mohamad Yazid bin Hossman[4], and Kanto Nishikawa[2,5]

[1]Department of Biology, Toho University, Funabashi, Chiba 274-8510, Japan
[2]Graduate School of Human and Environmental Studies, Kyoto University, Sakyo-ku, Kyoto 606-8501, Japan
[3]Chiba Biodiversity Center. Aoba-cho 955-2, Chuo-ku, Chiba 260-8682, Japan.
[4]Research, Development and Innovation Division, Sarawak Forest Department, Kuching 93250, Sarawak, Malaysia
[5]Graduate School of Global Environmental Studies, Kyoto University, Sakyo-ku, Kyoto 606-8501, Japan

*Corresponding author

Supplementary table S1. Data on feeding behaviour of the blunt-headed snail-eating snake, *Aplopeltura boa*.

Trial No.	Snake ID.	Duration (seconds)			Sawing	
		Extraction	Reposition	Sawing	No. of strokes	Side used
1	1	92	32	36	12	Right
2	1	264	80	117	22	Left
3	1	135	195	35	11	Right
4	2	52	223	44	25	Left
5	2	96	22	28	16	Right
6	2	97	41	56	27	Right
7	3	119	209	64	23	Right
8	4	134	459	41	6	Right
9	4	NA	NA	NA	NA	Left
10	4	109	73	30	6	Right
11	4	79	146	25	8	Right
12	4	NA	NA	NA	NA	Right
13	5	48	18	31	24	Right
14	5	36	36	15	11	Right
15	5	38	70	138	43	Left
16	5	NA	NA	NA	NA	Right
17	6	266	44	26	14	Right
18	6	115	66	26	14	Right
19	6	76	9	15	11	Right
20	6	108	41	23	8	Right
21	6	108	20	25	13	Right
22	6	106	9	27	14	Right
23	6	89	57	16	8	Left
24	6	68	11	20	12	Right
25	6	82	12	22	11	Right
26	7	57	114	29	12	Right
27	7	89	329	428	51	Right
28	8	126	115	40	16	Right
29	8	77	253	81	17	Left
30	8	274	113	53	18	Right
